# Generalized joint hypermobility does not influence 1-year patient satisfaction or functional outcome after ACL reconstruction

**DOI:** 10.1007/s00167-022-07008-0

**Published:** 2022-06-08

**Authors:** David Sundemo, Melker Svärd Jacobsson, Jón Karlsson, Kristian Samuelsson, Susanne Beischer, Roland Thomeé, Christoffer Thomeé, Eric Hamrin Senorski

**Affiliations:** 1 Department of Orthopedics, Institute of Clinical Sciences, Sahlgrenska Academy, University of Gothenburg, Göteborg, Sweden; 2 Department of Health and Rehabilitation, Institute of Neuroscience and Physiology, Sahlgrenska Academy, University of Gothenburg, Göteborg, Sweden; 3 Department of Orthopedics, Sahlgrenska University Hospital, Göteborg, Sweden; 4 Sportrehab Sports Medicine Clinic, Gothenburg, Sweden

**Keywords:** Anterior cruciate ligament, Anterior cruciate ligament reconstruction, Generalized joint hypermobility, Generalized joint laxity, Knee surgery, Sports medicine

## Abstract

**Purpose:**

The purpose of this study was to evaluate whether generalized joint hypermobility (GJH) influences postoperative results, including return to sport, patientreported outcomes, functional performance (hop tests), muscular strength, and the occurrence of ACL re-injury, in patients 1 year after anterior cruciate ligament (ACL) reconstruction.

**Methods:**

Data was extracted from a regional rehabilitation-specific registry containing information on patients with ACL injury. Patients between the ages of 16–50 years previously undergoing ACL reconstruction with available 1 year follow-up data were eligible for inclusion. Generalized joint hypermobility was assessed using the Beighton score (BS). Patients were examined one year postoperatively in terms of return to sport, patient-reported outcome, hop tests, muscular strength and the occurrence of reinjury. For purpose of analysis, patients were allocated into two groups, depending on the existence of GJH. The KOOS subscale of sports and recreation was considered the primary outcome. Analyses were performed both dichotomously and by using adjusted logistic regression, to consider potential confounders.

**Results:**

A total of 356 patients (41% males) were included, of which 76 (24% male) were categorized as having GJH. Patients with GJH had an inferior limb symmetry index preoperatively in terms of knee extension (mean 81.6 [SD 16.4] vs. 91.4 [SD 15.9], *p* = 0.02) and flexion strength (mean 91.9 vs. 99.1, *p* = 0.047) compared to patients without GJH. There was no difference between the groups in terms of the primary outcome, nor in any of the other postoperative outcomes. Nine patients (11.8%) in the group with GJH suffered ACL re-injury, compared with 13 patients (4.6%) in the control group (n.s.).

**Conclusion:**

One year after ACL reconstruction the existence of GJH did not affect postoperative patient satisfaction, strength or functional outcome. No conclusive statements can be made regarding the influence of GJH on the risk of ACL re-injury in this particular study.

**Level of evidence:**

Level II.

## Introduction

Generalized joint hypermobility (GJH) has been associated with an increased risk of anterior cruciate ligament (ACL) injury [12, 22, 23, 26]. Postoperative clinical outcomes in patients with an ACL injury with hypermobility have also been assessed [23]. Increased intermediate-term postoperative knee laxity has been observed, as well as inferior postoperative patient-reported outcome measurements, such as a poorer Lysholm score, International Knee Documentation Committee (IKDC) and Cincinnati knee rating system scores [13–15, 17]. Only one study has evaluated return to sport, using the Tegner Activity Scale, and it found no correlation with GJH. However, in that study, return to sport was evaluated as early as 6 months postoperatively, making it difficult to draw any definitive conclusions [1].

The primary purpose of this study was to evaluate whether GJH influences postoperative results relating to function on the sports and recreation subscale on the Knee injury and Osteoarthritis Outcome Score (KOOS) [20] in patients 1 year after anterior cruciate ligament reconstruction. The secondary purpose was to evaluate other postoperative parameters, such as return to sport, patient-reported outcome, recovery of functional performance, (hop tests) muscular strength and the occurrence of ACL re-injury. It was hypothesized that patients with GJH would have inferior results in terms of patient-reported outcomes, return to sport, functional performance and muscular strength. Moreover, it was hypothesized that GJH would increase the risk of ACL re-injury.

## Materials and methods

Patients recruited to a prospective rehabilitation registry (Project ACL registry) comprised the cohort in the present study. The Regional Ethical Review Board in Gothenburg granted ethical approval (265–13, T023-17). The Project ACL registry was initiated in September 2014 and is composed of two parts: (1) a web-based system used to acquire patient-reported outcome measurements and (2) a clinical part, comprising a battery of tests evaluating muscular strength and functional performance. To obtain data relating to concomitant injuries, the data on the patients in the cohort were linked to data from the Swedish Knee Ligament Registry.

Apart from assessing the strength and functional performance, the physical therapists enrolled in Project ACL also evaluated generalized joint hypermobility (GJH) using the Beighton Score (BS, Table 1) [3]. The assessment of the BS was introduced in January 2019. Prior to testing, all the involved physical therapists were trained in how to perform the BS evaluation to standardize the testing and improve inter- and intra-rater reliability. Previous studies have reported that the BS has acceptable reliability and validity [11]. One issue when evaluating the BS in patients with an ACL injury is the effect the traumatic injury may have on the degree of extension of the injured knee. This effect was resolved by using an injury allowance point [21]. The injury allowance point was attributed to patients with a positive hypermobility test on one side of a bilateral test but with a previous significant injury to the non-hypermobile joint. The injured joint was therefore assumed to have been equivalent, in terms of joint extension, to the contralateral joint at the pre-injury time point. In terms of the use of a BS cut-off, the guidelines presented at the latest consensus meeting, held in 2017 by the International Consortium of the Ehlers-Danlos Syndromes (ICEDS), were used [18]. The ICEDS implements individualized cut-offs depending on age and maturity, with a cut-off of ≥ 5 for pubertal men and women up to the age of 50. Since both boys and girls have experienced the last pubertal stage (Tanner stage V) at the age of 16 [6], patients aged between 16 and 50 were included in the study using the abovementioned cut-off value. Moreover, this age interval was selected, since the included population involves individuals in a physically active phase of life.

**Table 1:** Beighton hypermobility score

Maneuvers	Right side	Left side
Passive dorsiflexion of the fifth metacarpophalangeal joint to > 90°	1	1
Apposition of the thumb to the volar aspect of the ipsilateral forearm	1	1
Hyperextension of the elbow to > 10°	1	1
Hyperextension of the knee to > 10°	1	1
Palms placed flat on the floor without using knee flexion	1	

One point awarded for each maneuver, reaching a maximum of nine points. Tests are performed bilaterally and an injury allowance point.^35^ is used when scoring the injured knee

### Patients

**Fig. 1:**
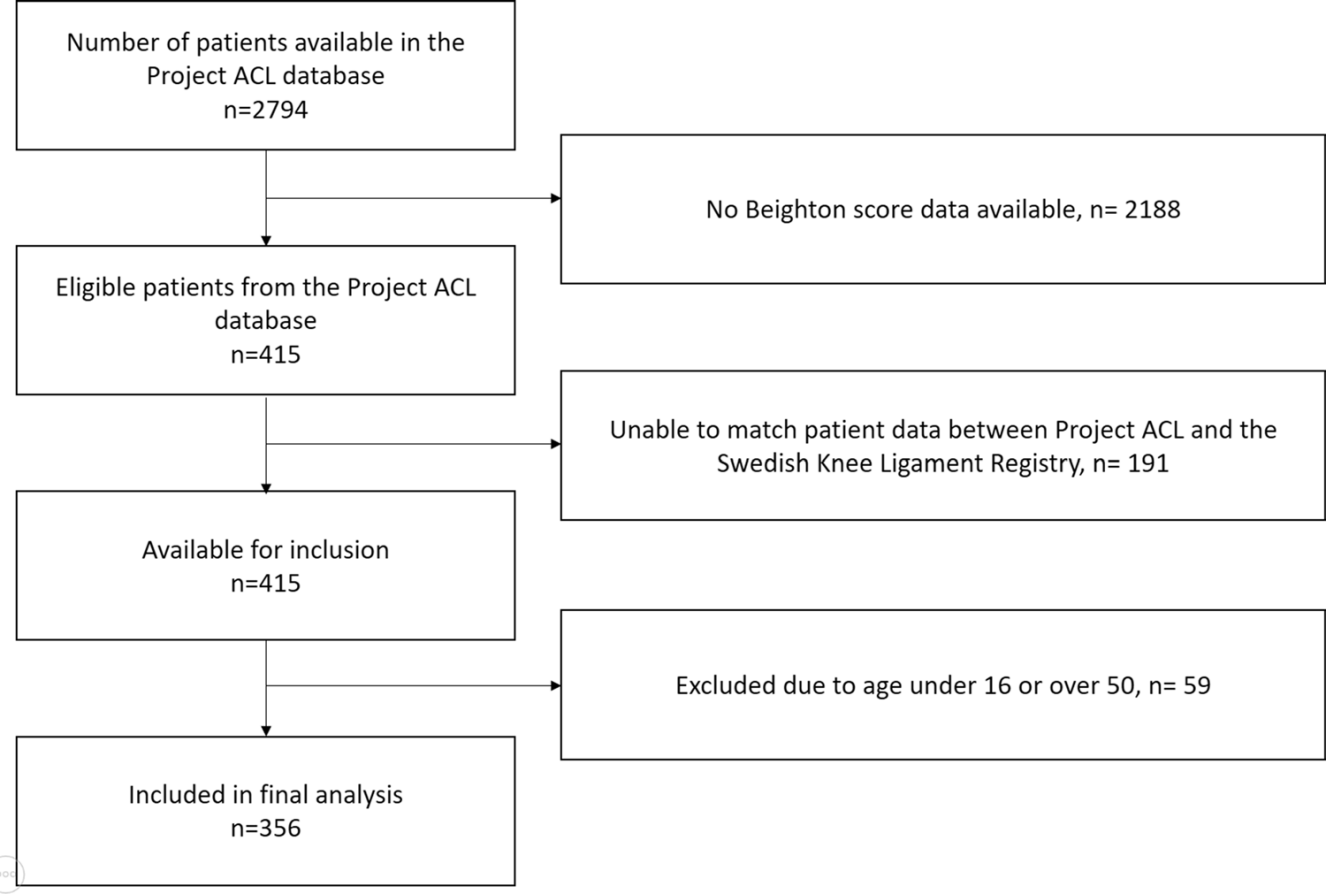
Flow chart of included patients. *ACL* anterior cruciate ligament

Patients between the ages of 16–50 years previously undergoing primary ACL reconstruction with available 1 year follow-up data in Project ACL were eligible for inclusion. Patients with previous ACL reconstruction or knee surgery were excluded. Finally, only patients with available data evaluating GJH registered in Project ACL were included. Patients received written information and written informed consent was obtained from all the patients (Fig. 1).

### Outcome

The primary outcome of the study was an evaluation of the function of the sports and recreation subscale on the KOOS [20]. Secondary outcomes involved the other KOOS subscales (evaluating pain, other symptoms, function in daily living, knee-related quality of life) and the Tegner Activity Scale [24]. Acceptable test–retest reliability has been reported for both the KOOS and the Tegner Activity Scale [5, 7].

Furthermore, isokinetic knee extension and flexion strength (Biodex Medical Systems, Shirley, New York) and hop tests (Table 2) were evaluated [4]. Three hop tests were evaluated in the following order: the vertical-hop test, the hop test for distance and the side-hop test (Table 2) [8, 19]. Both strength and hop tests were evaluated using the limb symmetry index (LSI), where the results for the injured limb are divided by the results for the contralateral healthy limb. This produces a quotient, indicating whether the individual performs symmetrically between the two injured limbs. An LSI above 90% is generally considered acceptable [2, 25]. Last, the incidence of ACL re-injury was considered. The parameter of ACL re-injury was defined as a subsequent ACL injury to either knee. The occurrence of ACL re-injury was diagnosed clinically and/or by use of magnetic resonance imaging.

**Table 2:** Tests of muscle function

	Degrees of movement	Practice trials	Test trials	Rest between repetitions, s	Units
Knee extension	90–0°	10 (50%) 10 (75%) 2 (90%)	3–4	40	Newton/meter
Knee flexion	0–90°	10 (50%) 10 (75%) 2 (90%)	3–4	40	Newton/meter
Vertical hop		2	3	20	Centimeters
Single-legged hop for distance		2	3–5	20	Centimeters
Side hop			30 s per side	180	Number of hops

S seconds, % represents the percentage of maximum force during the particular practice trial

### Statistical analysis

Statistical analysis was performed using SAS/STAT software (SAS Institute Inc, NC, USA). Baseline parameters were reported using the number and percentage for categorical variables. For continuous variables, the mean, standard deviation, median and range were presented. For ordered categorical variables, the median and range were presented. For between-group comparisons, the following methods were used: fisher’s exact test was used for dichotomous variables, the Mantel–Haenszel chi-square test was used for ordered categorical variables, the chi-square exact test was used for non-ordered categorical variables and Fisher’s non-parametric permutation test was used for continuous variables.

For comparisons between groups of the outcome variables at the one-year follow-up, Fisher’s non-parametric permutation test was used for continuous variables. The confidence interval for the mean difference between groups, presented in (Table 4), is based on Fisher’s non-parametric permutation test.

An à priori sample-size analysis was performed for the primary outcome. Assuming a group weight of 4:1, a standard deviation of 22.6 [9] (derived from the same cohort used in a previous study), a clinically significant difference in scores of 12.1 [10], an alpha of 0.05 and aiming for a power of 0.8, a total of 175 patients were needed to avoid a type-two error. A post-hoc power analysis was performed after observing the non-statistically significant but clinically important difference of 11.8 to 4.6% in the ACL re-injury rate. A power of 0.509 was found for this particular analysis. To generate power of 0.8, a total of 648 patients would have been required.

As a complement, univariable and adjusted linear regression analyses were performed on all the investigated outcomes. The analysis is presented using beta values, 95% confidence intervals, *p*-values and *R*-square numbers. The adjusted analysis was adjusted for factors which, according to the literature, are plausible confounders for the particular analyses. These factors were as follows: gender, age and type of graft.

## Results

A total of 356 patients met the inclusion criteria and had 1 year follow-up data available. A total of 76 (21.3%) patients fulfilled the criteria for GJH, 58 females (76,3% of all females) and 18 males (23,7% of all males, *p* = 0.0007). There were no differences in age, weight, height and BMI between groups. A semitendinosus tendon autograft was the most commonly used graft in both groups, 54 (72%) patients with GJH and 282 (81.7%) patients without received this option (Table 3). There were no differences in baseline preoperative KOOS and hop-test data between groups.

**Table 3:** Baseline parameters of included patients

	Total (*n* = 356)	Beighton score 0–4 (*n* = 280)	Beighton score 5–9 (*n* = 76)	*p*-value
Sex				
Female	210 (59%)	152 (54.3%)	58 (76.3%)	
Male	146 (41.0%)	128 (45.7%)	18 (23.7%)	0.0007
Age at index operation	25.9 (8.7) *n* = 356	25.8 (8.6) *n* = 280	26.3 (9.2) *n* = 76	n.s
Height [cm]	172.6 (9.3) *n* = 185	172.5 (9.1) *n* = 151	173.3 (10.6) *n* = 34	n.s
Weight [kg]	70.2 (11.1) *n *= 187	70.0 (11.0) *n* = 152	70.7 (11.7) *n* = 35	n.s
BMI [kg/m2]	23.5 (2.4) *n* = 185	23.4 (2.5) *n* = 151	23.6 (2.2) *n* = 34	n.s
Meniscus and/or cartilage injury				
No	143 (40.2%)	113 (40.4%)	30 (39.5%)	
Yes	213 (59.8%)	167 (59.6%)	46 (60.5%)	n.s
Medial meniscus injury				
No	275 (77.2%)	218 (77.9%)	57 (75.0%)	
Yes	81 (22.8%)	62 (22.1%)	19 (25.0%)	n.s
Lateral meniscus injury				
No	238 (66.9%)	183 (65.4%)	55 (72.4%)	
Yes	118 (33.1%)	97 (34.6%)	21 (27.6%)	n.s
Cartilage injury				
No	255 (71.6%)	200 (71.4%)	55 (72.4%)	
Yes	101 (28.4%)	80 (28.6%)	21 (27.6%)	n.s
Type of graft				
Patellar tendon	61 (17.2%)	42 (15.1%)	19 (25.3%)	
Hamstring tendon	282 (79.7%)	228 (81.7%)	54 (72.0%)	
Quadriceps tendon	8 (2.3%)	6 (2.2%)	2 (2.7%)	
Allograft	2 (0.6%)	2 (0.7%)	0	
Direct-suture/synthetic/other	1 (0.3%)	1 (0.4%)	0	n.s
Missing	2	1	1	
Patient-reported outcome				
KOOS sports and recreation	39.4 (25.4) *n* = 105	40.0 (25.0) *n* = 84	36.7 (27.1) *n* = 21	n.s
KOOS pain	73.2 (16.3) *n* = 105	73.5 (17.0) *n* = 84	72.0 (13.0) *n* = 21	n.s
KOOS symptoms	66.6 (18.3) *n* = 105	66.3 (18.7) *n* = 84	67.8 (17.3) *n* = 21	n.s
KOOS daily living	85.4 (15.3) *n* = 104	85.3 (16.2) *n* = 83	85.6 (11.2) *n* = 21	n.s
KOOS quality of life	35.5 (18.6) *n* = 105	35.8 (18.1) *n* = 84	34.0 (20.9) *n* = 21	n.s
Hop tests				
LSI single-leg hop test	86.0 (14.7) *n* = 12	85.2 (15.1) *n* = 11	95.6 *n* = 1	n.s
LSI vertical jump test	80.4 (16.5) *n* = 14	80.7 (17.1) *n* = 13	76.5 *n* = 1	n.s
LSI side-hop test	90.0 (14.4) *n* = 6	92.7 (14.2) *n* = 5	76.3 *n* = 1	n.s
Muscular strength				
LSI Knee extension strength	89.2 (16.4) *n* = 89	91.4 (15.9) *n* = 69	81.6 (16.4) *n* = 20	0.020
LSI Knee flexion strength	97.5 (13.7) *n* = 89	99.1 (13.5) *n* = 69	91.9 (13.3) *n* = 20	0.047

For categorical variables, *n* (%) is presented

For continuous variables, the mean (SD) and *n* are presented. For comparisons between groups, Fisher’s exact test (lowest one-sided *p*-value multiplied by 2) was used for dichotomous variables, the Mantel–Haenszel chi-square test was used for ordered categorical variables, the chi-square exact test was used for non-ordered categorical variables and Fisher’s non-parametric permutation test was used for continuous variables

*KOOS* knee injury and osteoarthritis outcome score, *LSI* limb symmetry index

Preoperatively, patients with GJH obtained an inferior LSI score in terms of knee extension (mean 81.6 vs. 91.4, *p* = 0.02) and knee flexion strength (mean 91.9 vs. 99.1, *p* = 0.047) compared with patients without GJH (Table 3).

### Postoperative outcome

One year after ACL reconstruction, there was no difference between the groups in terms of the primary outcome: the KOOS function on the sport and recreation subscale (Table 4). No differences were found regarding the other KOOS subscales or for the postoperative physical activity level, measured using the Tegner Activity Scale. The analysis of the hop tests did not identify any differences between the groups. The mean LSI was above 90% for all the hop tests. The differences in knee flexion and extension strength observed between the groups preoperatively did not remain postoperatively (Table 4). The complementary adjusted linear regression analysis did not indicate any influence of GJH on any of the investigated outcomes (Table 5).

**Table 4:** Dichotomous analysis of investigated outcomes one year after ACL reconstruction

Outcome parameters at 1 year postoperatively	Total (*n* = 356)	Beighton score 0–4 (*n* = 280)	Beighton score 5–9 (*n* = 76)	*p*-value
Patient-reported outcome				
KOOS sports and recreation	68.7 (22.8) *n* = 194	69.5 (22.9) *n* = 151	65.8 (22.6) *n* = 43	n.s
KOOS pain	86.8 (11.5) *n* = 194	87.2 (11.1) *n* = 151	85.4 (12.9) *n* = 43	n.s
KOOS symptoms	77.9 (14.9) n = 194	78.3 (15.3) n = 151	76.7 (13.5) n = 43	n.s
KOOS daily living	95.1 (7.8) *n* = 194	95.2 (7.9) *n* = 151	94.4 (7.5) *n* = 43	n.s
KOOS quality of life	58.9 (18.4) *n* = 194	59.2 (18.5) *n* = 151	57.6 (18.3) *n* = 43	n.s
Tegner activity scale	5.44 (1.00; 10.00) *n* = 199	5.47 (1.00; 10.00) *n* = 154	5.31 (1.00; 9.00) *n *= 45	n.s
Hop tests				
LSI single-legged hop test	95.0 (11.1) *n* = 165	94.9 (11.0) *n* = 135	95.2 (11.6) *n* = 30	n.s
LSI vertical jump test	90.4 (16.5) *n* = 167	90.2 (15.7) *n* = 137	91.0 (19.8) *n* = 30	n.s
LSI side-hop test	96.9 (18.2) *n* = 151	97.3 (18.2) *n* = 125	94.7 (18.4) *n* = 26	n.s
Muscular strength				
LSI Knee extension strength	93.0 (14.4) *n* = 191	93.4 (14.6) *n* = 153	91.3 (13.8) *n* = 38	0.42
LSI Knee flexion strength	98.7 (11.0) *n* = 191	98.9 (11.0) *n* = 153	97.8 (11.0) *n* = 38	n.s
ACL re-injury within 12 months				
No re-injury	334 (93.8%)	267 (95.4%)	67 (88.2%)	
Re-injury (12 m)	22 (6.2%)	13 (4.6%)	9 (11.8%)	n.s

For continuous variables, the mean (SD) and *n* is presented. For comparisons between groups, Fisher’s non-parametric permutation test was used for continuous variables. The confidence interval for the mean difference between groups is based on Fisher’s non-parametric permutation test

*CI* confidence interval, *KOOS* knee injury and osteoarthritis outcome score, *LSI* limb symmetry index

**Table 5:** Linear regression analysis assessing the influence of generalized joint hypermobility on the investigated outcomes

Outcome parameters one year postoperatively	Univariable^a^			Adjusted^b^	
	Beta (95% CI)	*p*-value	*R*-Square	Beta (95% CI)	*p*-value
Patient-reported outcome					
KOOS sports and recreation	− 0.64 (− 2.25;0.97)	0.43	0.00	− 0.86 (− 2.51;0.78)	n.s
KOOS pain	− 0.33 (− 1.14;0.48)	0.43	0.00	− 0.36 (− 1.21;0.49)	n.s
KOOS symptoms	− 0.13 (− 1.18;0.92)	0.81	0.00	− 0.26 (− 1.35;0.84)	n.s
KOOS daily life	− 0.04 (− 0.59;0.51)	0.88	0.00	− 0.14 (− 0.72;0.43)	n.s
KOOS quality of life	− 0.04 (− 1.33;1.26)	0.96	0.00	− 0.32 (− 1.67;1.02)	n.s
Tegner activity scale	− 0.03 (− 0.20;0.13)	0.69	0.00	− 0.03 (− 0.19;0.13)	n.s
Hop tests					
LSI single-legged hop test	0.14 (− 0.73;1.01)	0.75	0.00	− 0.05 (− 0.93;0.83)	n.s
LSI vertical jump	0.47 (− 0.82;1.76)	0.47	0.00	0.21 (− 1.08;1.50)	n.s
LSI side-hop test	0.06 (− 1.49;1.61)	0.93	0.00	0.11 (− 1.48;1.71)	n.s
Muscular strength					
LSI quadriceps strength	− 0.08 (− 1.13;0.97)	0.88	0.00	0.03 (− 0.99;1.05)	n.s
LSI hamstring strength	− 0.30 (− 1.10;0.49)	0.45	0.00	− 0.46 (− 1.25;0.32)	n.s

*CI* confidence interval, *KOOS* knee injury and osteoarthritis outcome score, *LSI* limb symmetry index

*P*-values, beta and R-square are based on original values and not on stratified groups

^a^All tests are performed with univariable linear regression

^b^Adjusting for sex, graft (surgery) and age at index operation using linear regression

The ACL re-injury rates within one year after ACL reconstruction did not show any statistically significant difference (11.8 vs. 4.6%, n.s.). Considering the clinically important difference of 11.8 to 4.6% in ACL re-injury rate, a post-hoc power analysis was performed. It demonstrated a power of 0.509 for this particular analysis. To generate a power of 0.8, a total of 648 patients would have been required.

## Discussion

The principal finding in this study was that there was no difference in terms of sports-related patient-reported outcomes with regard to the existence of GJH one year after ACL reconstruction. These results contradict the hypothesis and they may appear to be in contrast to those of previous studies evaluating patient-reported outcomes for patients with an ACL reconstruction [17]. Larson et al. [17] reported that patients with GJH had inferior IKDC, Cincinnati and Lysholm scores at a mean of 6 years postoperatively. Moreover, using group comparisons, Kim et al. [13, 14] demonstrated that patients with GJH had an inferior Lysholm score 2, 5 and 8 years after ACL reconstruction compared with controls with no GJH. It may be that there are no obvious short-term negative effects for individuals with GJH and this would not have been identified in previous studies since they used a longer follow-up period (2, 5, 6 and 8 years) [13, 14, 17]. Future follow-ups of the current cohort of patients with GJH, using longer observation times, will reveal whether these acceptable results remain or whether these patients with GJH tend to deteriorate more rapidly than their non-hypermobile peers beyond one year after ACL reconstruction, as can be observed elsewhere in the literature.

Another interesting observation was the incidence of ACL re-injury in patients with GJH. Nine patients (11.8%) in the group with GJH suffered ACL re-injury, compared with 13 patients (4.6%) in the control group (n.s.). The results were not statistically significant and no conclusions can therefore be drawn regarding the influence of GJH on ACL re-injury risk. However, considering the substantial difference in incidence, purpose-built studies with sufficient statistical power must clearly be designed to answer this important question conclusively. In contrast to the current study, a systematic review investigating the incidence of graft rupture in patients with previous ACL reconstruction written by Krebs, Barber-Westin and Noyes concluded that patients with GJH run an increased risk of ACL re-injury [16]. The current study is by far the largest study available assessing the risk of ACL re-injury in patients with GJH. Nevertheless, the study lacks statistical power and, as a result, no definitive conclusions regarding ACL re-injury can be drawn.

There are other interesting findings in the current study. Comparisons between groups in terms of hop performance and muscle strength were not directly evaluated, but the difference in relative performance between the injured and non-injured limbs was instead investigated. Patients with GJH had a lower LSI preoperatively in terms of both quadriceps and hamstring strength compared with patients without GJH. This could possibly be a factor contributing to the higher risk of ACL rupture among patients with GJH. Identifying muscle asymmetry in individuals with GJH, before injury, could possibly prevent a potential future ACL injury by giving these patients specific training programs. However, further studies investigating the existence of preoperative asymmetry are needed. Interestingly, postoperative mean and median levels of LSI above 90%, generally regarded as acceptable [2, 25], were reached on average in both strength and hop tests for both subgroups. Acceptable results are therefore to be expected, in terms of muscular strength and hop-test rehabilitation, regardless of the presence of GJH, at least in the short term.

There are a few limitations to the present study that warrant discussion. First, it was evident that the study was underpowered in terms of the analysis of one of the secondary endpoints, the analysis of ACL re-injury, not reaching the preferred 0.8 power limit. Second, the use of the LSI in interpreting the rehabilitation of knee function is not entirely unproblematic. A previous study concluded that it may be more appropriate to use the results for the uninjured knee, early post-injury, as a benchmark for the rehabilitation of the injured knee [27]. The strength and function of the uninjured knee deteriorate post-injury, where instability and pain impair knee mobility and strength. So, if they are unaware of the shortcomings of the LSI, readers may assume that an LSI close to 100% suggests the achievement of preinjury strength in the injured knee, which is not necessarily the case.

## Conclusion

One year after ACL reconstruction, the existence of GJH did not affect postoperative patient satisfaction, strength or functional outcome. No conclusive statements can be made regarding the influence of GJH on the risk of ACL re-injury in this particular study.
